# Effects of thrombolysis within 6 hours on acute cerebral infarction in an improved rat embolic middle cerebral artery occlusion model for ischaemic stroke

**DOI:** 10.1111/jcmm.14120

**Published:** 2019-01-29

**Authors:** Zhihua Si, Jinzhi Liu, Ke Hu, Yan Lin, Jie Liu, Aihua Wang

**Affiliations:** ^1^ Department of Neurology Shandong Provincial Qianfoshan Hospital Affiliated to Shandong University Jinan Shandong China; ^2^ Department of Emergency Qianfoshan Hospital Affiliated to Shandong University Jinan China; ^3^ Department of Internal Medicine Shandong Provincial Police General Hospital Jinan Shandong China; ^4^ Department of Neurology People's Hospital of Rizhao Rizhao Shandong China

**Keywords:** cerebral infarction, recombinant tissue plasminogen activator, stroke, thrombolysis

## Abstract

Recombinant tissue plasminogen activator (rt‐PA) is the first‐line drug for revascularization in acute cerebral infarction (ACI) treatment. In this study, an improved rat embolic middle cerebral artery occlusion model for ischaemic stroke was used and the rats were killed on the first, third and seventh day after model establishment. Increases in infarct volume were significantly less in the thrombolytic group than in the conventional group at every time‐point. The microvascular density (MVD) in the thrombolytic group was significantly higher than that in the conventional group at every time‐point, especially on the seventh day. Increases in the expressions of neuronal nitric‐oxide synthase (NOS) and caspase‐3 in the ischaemic region and in the nitric oxide contents, malondialdehyde contents, and inducible NOS activities in the cortex of infarct side were significantly less in the thrombolytic group than in the conventional group. Furthermore, decreases in the superoxide dismutase activities in the thrombolytic group were significantly less than those in the conventional group. In conclusion, thrombolytic rt‐PA therapy within a broadened therapeutic window (6 hours) could significantly decrease the infarct volume after ACI, possibly by increasing MVD in the ischaemic region, decreasing apoptotic molecule expression, and alleviating the oxidative stress response.

## 1 INTRODUCTION

Stroke, one of the most common diseases that affect human health, is the second leading cause of mortality and the first leading cause of disability in China.^1^ Recombinant tissue plasminogen activator (rt‐PA) was first reported to be effective in the treatment of acute cerebral infarction (ACI) patients in 1995.^2^ A recent study showed that rt‐PA is still the first‐line drug for revascularization in ACI treatment.^3^ However, because of the limitation of a 4.5 hours thrombolysis time window,^4^ most patients do not have the opportunity to receive such therapy. Therefore, effective measures in the treatment of acute ischaemic stroke are needed.

In recent years, it has been reported that the thrombolysis of ACI patients could be performed 6 hours after ACI.^5^ Recent study demonstrated that thrombolytic therapy 3‐9 hours after ACI could dramatically improve magnetic resonance imaging or computerized tomography results as well as clinical outcome.^6^ However, the mechanism of thrombolytic therapy within a broadened therapeutic window is less obvious.

Neovascularization is a key player in ischaemic neural survival after ACI,^7^ which is critical for the recovery of learning and memory abilities and for improved prognosis. Ischaemia and reperfusion can lead to a significant increase in free radicals and lipid peroxidation, thereby exacerbating brain damage.^8^, ^9^, ^10^, ^11^, ^12^ The excessive release of nitric oxide (NO), an active gas free radical, could lead to neuron damage.^13^ Malondialdehyde (MDA), the degradation product of lipid peroxidation, also causes direct damage to the brain.^14^ Superoxide dismutase (SOD), a critical antioxidant enzyme in brain tissues, plays an important role in cerebral protection by eliminating excessive intracellular free radicals.^15^ Therefore, these molecules might be key regulators implicated in the effects of rt‐PA on ACI.

In this study, we used an improved rat embolic middle cerebral artery occlusion (MCAO) model to study the effects of thrombolytic therapy with rt‐PA 6 hours after MCAO on infarct volume, microvascular density (MVD), caspase‐3, nitric oxide , nitric‐oxide synthase (NOS), MDA and SOD in the ischaemic region after ACI in an aim to uncover the underlying mechanism of thrombolytic therapy within a broadened therapeutic window and thus to provide a reliable theory basis for the clinical treatment of ACI.

## 2 MATERIALS AND METHODS

### 2.1 Animals

This study was approved by the Ethics Committee of Shandong Provincial Qianfoshan Hospital, China, and all experiments were performed in accordance with the hospital's relevant guidelines and regulations.

One hundred and 62 adult male Sprague‐Dawley rats (320‐350 g), aged 2 months, were provided by the Experimental Animal Center, Shandong University of Traditional Chinese Medicine, Jinan, China. All rats were housed in a temperature‐controlled (22 ± 2°C) and humidity‐controlled (58%‐68%) room under an 8:16 hours light cycle. They were equally and randomly divided into three groups (54 rats per group): the sham group, the conventional group (i.e. the conventionally treated infarction group) and the thrombolytic group (i.e. the infarction group treated with rt‐PA 6 hours after ACI). Each group was further randomly and equally divided into three subgroups (18 rats per group) depending on the days after model establishment (first, third, or seventh day).^16^, ^17^, ^18^, ^19^

### 2.2 Middle cerebral artery occlusion model

An improved rat MCAO model that was embolized by autologous blood clots was established as follows: (a) *Preparation of thrombus*: 0.6 mL venous blood drawn from a caudal vein and 0.15 mL thrombin (200 U/mL) were mixed, quickly placed into PE50 tubes, and allowed to set for 4 hours. The thrombus was cut into small emboli (1 mm) and placed into PE50 tubes for backup. (b) *Embolic MCAO model establishment*: The rats were first anaesthetized with an intraperitoneal injection of 6% chloral hydrate (35 mg/100 g). A 2‐cm‐long midline incision on the neck was made, and the right common carotid artery (CCA), external carotid artery (ECA), internal carotid artery (ICA), occipital artery (OA), superior thyroid artery (STA) and pterygopalatine artery (PPA) were then dissected. The OA, STA, PPA and the distal end of ECA were ligatured, and the CCA and ICA were temporarily clipped. A PE‐50 tube containing blood clots was inserted into the proximal end of the ECA, and the microvascular clips were then opened to allow the clot infusion (10‐12 emboli). The clips of CCA were removed, and the incision was sutured. (c) *Intervention*: The conventional group was intraperitoneally injected with citicoline (500 mg/kg, once a day; Shandong Qilu Pharmaceutical Factory, Shandong, China) 24 hours after the MCAO^20^; the thrombolytic group, on the basis of the above medications, was treated with an intravenous injection of rt‐PA (10 mg/kg) 6 hours after the MCAO; the same procedures were performed in the sham group except for no clot infusion and intravenous injection of the vehicle saline. A grading scale of 0‐4 was performed to access the neurological function deficit scale after model establishment, as previously described^21^: 0—no obvious neurological function deficit; 1—mild degree of neurological dysfunction (i.e. when raising the rat by its tail, the contralateral forelimb cannot be fully extended); 2—moderate degree of focal neurological dysfunction (i.e. rotate to the right when walking); 3—severe degree of focal neurological dysfunction (i.e. dump to the right when walking); 4—unable to walk or in coma. Rats showing a score of 1‐3 were considered to be infarcted.

### 2.3 2,3,5‐triphenyl tetrazolium chloride staining

Six rats from each of above three groups were randomly selected on the first, third, and seventh day after model establishment. The rats were deeply anaesthetized and killed. The brains were rapidly removed, coronally sectioned at 2 mm, and stained with 2% 2,3,5‐triphenyl tetrazolium chloride (Sigma, San Louis, MO, USA) in 0.9% saline for 30 minutes at 37°C in darkness. The slices were fixed in 10% neutral formalin overnight, and pictures were then taken using a digital camera. Image analysis was performed with Image J 1.41 software. The infarct size was normalized for oedema. The infarct volume for each brain was calculated as *I*% = (volume of contralateral−normal volume of ipsilateral)/volume of the contralateral brain.

### 2.4 Immunohistochemistry

Six rats from each of above three groups were randomly selected on the first, third and seventh day after model establishment. The animals were killed and perfused with 0.9% saline followed by 4% paraformaldehyde perfusion. The brains were rapidly removed, fixed with 4% paraformaldehyde for 24 hours, and embedded in paraffin. Continuous cryosections (3 μm) were made and examined by immunohistochemistry according to the instructions of the manufacturer (Chinese Fir Golden Bridge Company, Beijing, China). The primary antibodies used were rabbit anti‐rat CD34 antibody (1:100, ab81289; Abcam, Cambridge, MA, USA), anti‐nNOS antibody (1:100, ab5586; Abcam), and anti‐caspase‐3 antibody (1:50, ab13847; Abcam). The primary antibodies were detected with the anti‐rabbit biotinylated secondary antibody (1:200, PK‐4001; Chinese Fir Golden Bridge Company). Phosphate‐buffered saline was used for negative control. Diaminobenzidine was used to visualize the reaction.

The MVD, nNOS‐positive cells and caspase‐3‐positive cells of the ischaemic region in the infarction group were observed under a microscope (400 X). Five fields per slice were randomly selected for the microvessel or positive cell number counting, and the mean served as the MVD or the expression of nNOS or caspase‐3 of this slice. The mean of microvessel or positive cell number count from all the slices in one group served as the MVD, or expression of nNOS or caspase‐3 of this group.

### 2.5 Determination of nitric oxide and MDA contents and iNOS and SOD activities

Six rats from each of above three groups were randomly selected on the first, third and seventh day after model establishment. The animals were killed, and the brains were rapidly removed. The infarct‐side cortexes were rapidly frozen in liquid nitrogen and then stored at −80°C. The cortex tissue samples were homogenized in cold saline to make 10% homogenates. They were then centrifuged at 1248 *g*, and the supernatants were collected and stored at −80°C. Nitric oxide and MDA contents and iNOS and SOD activities were measured according to the manufacturer's instructions using the nitric oxide assay kit (A013‐2; Nanjing Jiancheng Biology Engineering Institute, Nanjing, China), MDA assay kit (TBA method) (A003‐1; Nanjing Jiancheng Biology Engineering Institute), NOS typed assay kit (Colormetric method) (A014‐1‐1; Nanjing Jiancheng Biology Engineering Institute), and SOD assay kit (WST‐1 method) (A001‐3; Nanjing Jiancheng Biology Engineering Institute), respectively.

### 2.6 Statistical analysis

All the statistical analyses were performed with SPSS. The data in accordance with normal distribution and homogeneity of variance were presented as mean ± SD. Data between the groups were analysed by one‐way analysis of variance, followed by Fisher's protected least significant difference post‐hoc tests. Statistically significant differences were defined as *P *< 0.05.

## 3 RESULTS

### 3.1 Thrombolysis decreased the infarct volume after the MCAO

**Figure 1:**
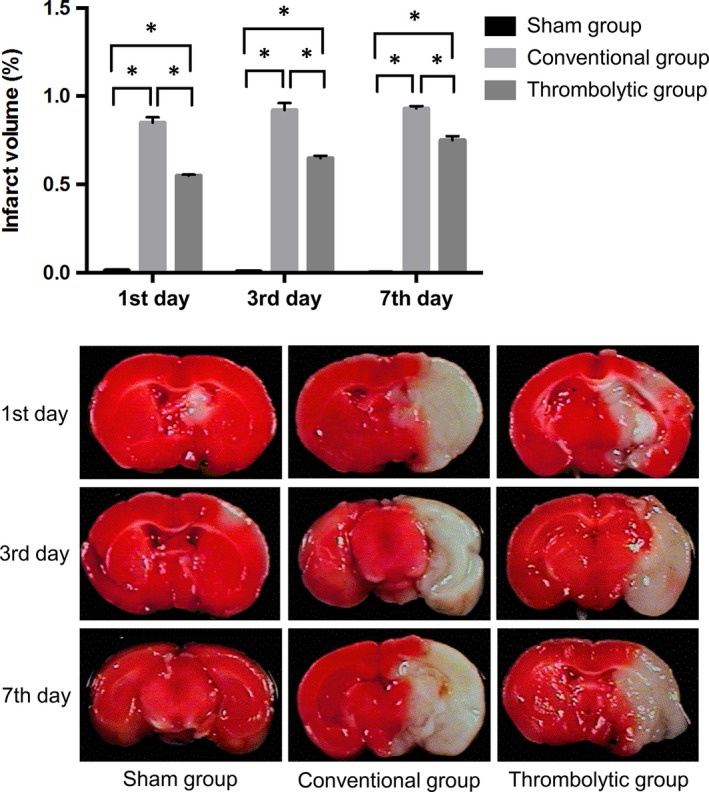
Thrombolysis decreased the infarct volume after middle cerebral artery occlusion. Upper: Quantification of infarct volume obtained from 2,3,5‐triphenyl tetrazolium chloride (TTC) staining in the sham group, conventional group and thrombolytic group. * indicates *P *<* *0.05. Lower: Representative images of TTC staining in the above groups

No obvious infarct region was observed in the sham group, and an obvious infarct region, (white colour) was seen in the infarction groups. The infarct volume was significantly higher in the conventional group than in the sham group at every time‐point (0.85 ± 0.031% vs 0.015 ± 0.00% on the 1st day; 0.92 ± 0.040% vs 0.008 ± 0.00% on the 3rd day; 0.93 ± 0.014% vs 0.0025 ± 0.00% on the 7th day; all *P* < 0.05). Thrombolysis could potently decrease the infarct volume at every time‐point relative to the conventional group (0.55 ± 0.006% vs 0.85 ± 0.031% on the 1st day; 0.65 ± 0.013% vs 0.92 ± 0.040% on the 3rd day; 0.75 ± 0.024% vs 0.93 ± 0.014% on the 7th day; all *P* < 0.05) (Figure 1).

### 3.2 Thrombolysis increased the MVD after the MCAO

**Figure 2:**
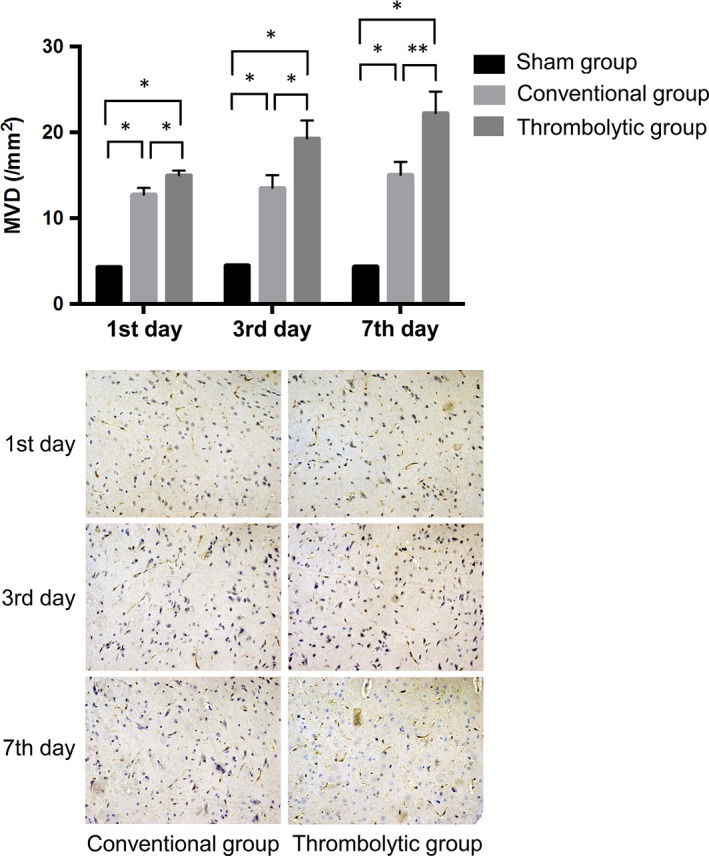
Thrombolysis increased the microvascular density (MVD) after MCAO. Upper: Quantification of MVD obtained from immunohistochemistry in the sham group, conventional group and thrombolytic group. * indicates *P *<* *0.05; ** indicates *P *<* *0.01. Lower: Representative images of MVD obtained from immunohistochemistry in the conventional group and thrombolytic group under a microscope (400X)

There were no changes in the MVD in the sham group at any time‐point. The MVD expression in the infarction groups, especially the thrombolytic group, was significantly elevated at every time‐point (*P *<* *0.05). Furthermore, the MVD expression was significantly higher in the thrombolytic group than that in the conventional group at all time‐points (*P < *0.05), especially on the 7th day (*P < *0.01) (Figure 2).

### 3.3 Thrombolysis decreased the expression of nNOS and caspase‐3 after the MCAO

**Figure 3:**
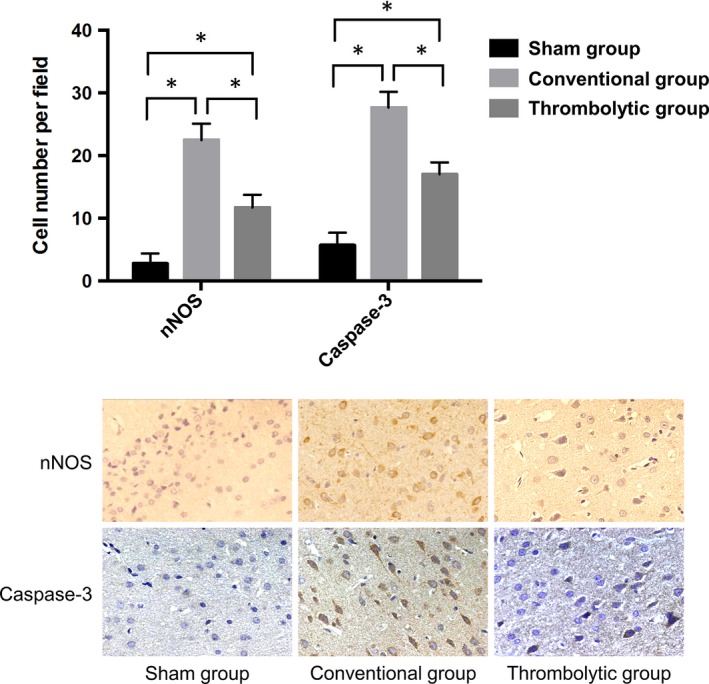
Thrombolysis decreased the expression of nNOS and caspase‐3 after MCAO. Upper: Quantification of nNOS and caspase‐3 expression obtained from immunohistochemistry in the sham group, conventional group, and thrombolytic group. * indicates *P *<* *0.05. Lower: Representative images of nNOS and caspase‐3 expression obtained from immunohistochemistry in the above groups under a microscope (400 X)

The nNOS‐positive cells and caspase‐3‐positive cells were stained with brown or tan. There were few positive cells in the sham group. One day after the MACO, the expressions of nNOS and caspase‐3 in the ischaemic region were enhanced in the conventional group compared with that of the sham group (*P < *0.05). Furthermore, their expression was decreased in the thrombolytic group compared with the conventional group (*P < *0.05). (Figure 3).

### 3.4 Thrombolysis decreased the free radical activities and increased SOD activities after the MCAO

**Figure 4:**
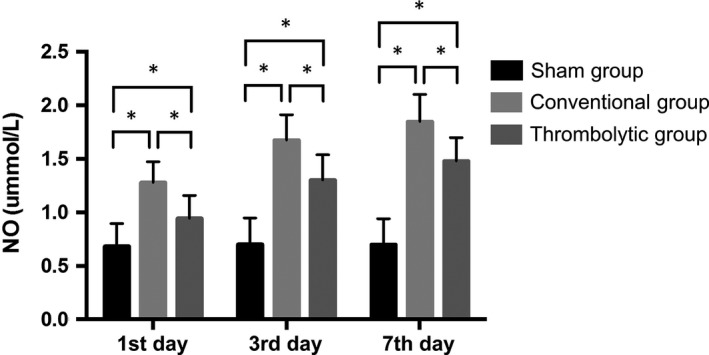
Thrombolysis decreased nitric‐oxide (NO) contents after MCAO. Quantification of nitric oxide contents in the sham group, conventional group and thrombolytic group. * indicates *P *<* *0.05

**Figure 5:**
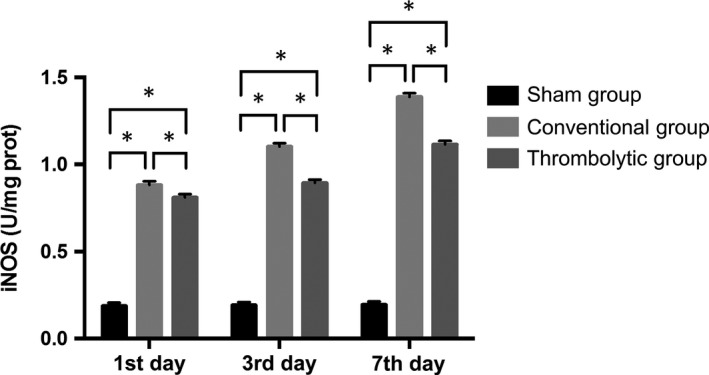
Thrombolysis decreased nitric‐oxide activities after MCAO. Quantification of iNOS activities in the sham group, conventional group and thrombolytic group. * indicates *P *<* *0.05

**Figure 6:**
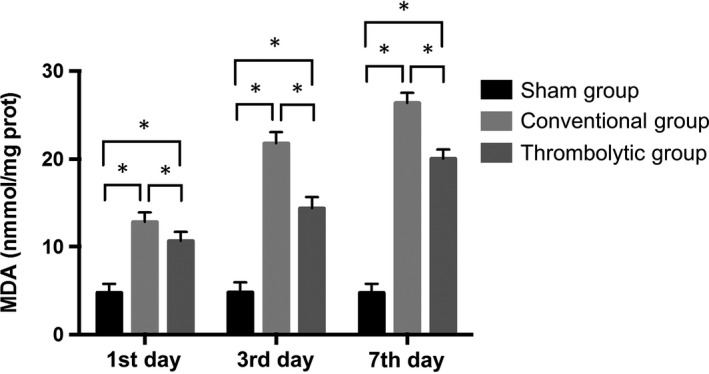
Thrombolysis decreased malondialdehyde (MDA) contents after MCAO. Quantification of MDA contents in the sham group, conventional group and thrombolytic group. * indicates *P *<* *0.05

**Figure 7:**
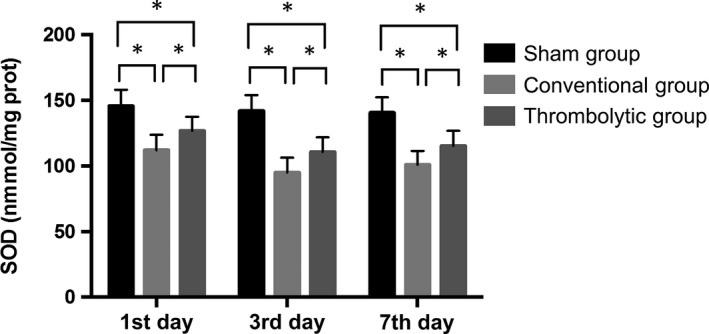
Thrombolysis increased superoxide dismutase (SOD) activities after MCAO. Quantification of SOD activities in the sham group, conventional group, and thrombolytic group. * indicates *P *<* *0.05

Nitric oxide and MDA contents and iNOS activities were potently increased in the cortex of the infarct side relative to the sham group at all time‐points (*P < *0.05) and they were significantly decreased by thrombolytic treatment compared with the conventional group (*P < *0.05). In contrast, the SOD activities were decreased in the cortex of the infarct side relative to the sham group at all time‐points (*P < *0.05), and they were dramatically increased in the thrombolytic group compared with the conventional group (*P < *0.05). (Figures 4, 5, 6, 7).

## 4 DISCUSSION

Clinical practice has proved that the early thrombolysis of ACI could dramatically improve the prognoses and life quality of patients. At present, the intravenous infusion of rt‐PA within 3 h is the most efficient therapy for ACI.^22^ However, the use of rt‐PA is limited by its narrow therapeutic window. Therefore, the extent to which more patients can benefit from thrombolytic therapy remains to be studied. A recent study demonstrated that the thrombolytic therapy 3‐9 hours after ACI could significantly improve the clinical outcome of patients^6^; however, the underlying mechanism is largely unknown. In our study, we used an improved rat embolic MCAO model and found that rt‐PA treatment 6 hours after ACI could significantly decrease infarct volume, which was consistent with previous studies.^23^

Neovascularization is a key player in ischaemic neural survival after ACI,^7^ which is critical for promoting the recovery of learning and memory abilities and for improving prognosis. The improvement of cerebral blood flow is directly related to the extent of microangiogenesis. It has been reported that ischaemia could induce VEGF expression in macrophages, astrocytes and neurons to promote neovascularization, and thus increase the oxygen and blood supply for infarct periphery and reduce the infarct size.^23^, ^24^ Consistent with these findings, our study showed that the MVD was significantly enhanced after the MCAO, especially in the thrombolytic group. The MVD in the thrombolytic group was significantly higher than that in the conventional group at every time‐point (*P* < 0.05), especially on the seventh day (*P* < 0.05). Taken together, these results indicate that as the duration of hypoxia in the ischaemic region is extended, the MVD is increased as a compensatory result and is further promoted by thrombolysis.

In cerebral ischaemia‐reperfusion injury, oxidative stress is a major mechanism that aggravates the brain damage.^8^, ^9^, ^10^ Ischaemia and reperfusion can lead to a surge in free radicals, and the biosynthesis of nitric oxide is a key player in the pathophysiological response.^13^ Nitric oxide, synthesized by NOS from l‐arginine, is a very active gas free radical. Three types of NOS in biological organisms have been identified: nNOS, endothelial NOS (eNOS) and iNOS. Nitric oxide plays an important role in message transmission and neuroprotection in the central nervous system, but its excessive release could lead to neuron damage. The neurotoxicity of nitric oxide results mainly from its reaction with oxygen free radicals, thus producing ONOO‐, NO2· and OH·. In addition, nNOS‐derived endogenous nitric oxide has also been reported to play a detrimental role in the process of cerebral ischaemia‐reperfusion.^25^ In the present study, compared with the sham group, all of nitric oxide contents and NOS expression and activities were significantly increased in the infarct‐side cortexes at all time‐points (*P* < 0.05), and they were significantly decreased by thrombolytic treatment compared with the conventional group (*P* < 0.05), indicating that thrombolysis with rt‐PA 6 hours after ACI still has clinical significance. A significant finding is that no increase in mortality was found in elderly patients with thrombolytic treatment 6 hours after stroke.^26^, ^27^ Taken together, these results suggest that thrombolysis 6 hours after ACI could suppress nitric oxide‐induced oxidative stress injury and neurotoxicity to improve patient outcome.

In addition, ACI‐reperfusion leads to lipid peroxidation.^11^, ^12^ MDA, the degradation product and the index of lipid peroxidation, has a very strong biological toxicity and causes direct damage to thebrain.^14^ SOD, a critical antioxidant enzyme in brain tissues, plays an important role in cerebral protection by eliminating excessive intracellular free radicals.^15^ Our results showed that the MDA contents and SOD activities were significantly decreased and increased, respectively, in the thrombolytic group at all time‐points compared with the conventional group (*P* < 0.05). These results further support our hypothesis that thrombolysis within 6 hours after ACI could suppress oxidative stress injury and neurotoxicity to improve patient outcome.

Cerebral ischaemia was first reported to cause neural apoptosis in 1990^28^; since then, numerous studies have been conducted to explore the underlying mechanisms and therapeutic treatments. Recent studies have shown that cell apoptosis is a crucial part of cerebral ischaemia and a major mechanism responsible for secondary brain damage after cerebral ischaemia.^29^ Cerebral ischaemia induces a rapid death in the cells of the ischaemic central region, and a delayed death in the cells of the ischaemic penumbra region by causing cell apoptosis.^30^ Cell apoptosis after cerebral ischaemia‐reperfusion results from many factors. The activation of caspase‐3 could cause multiple gene changes that degrade and deactivate key proteins of cytoplasm, the nucleus, and the cytoskeleton, leading to irreversible cell apoptosis. Our findings showed that caspase‐3 was activated in infarction groups, and its expression was decreased in the thrombolytic group compared with in the conventional group (*P* < 0.05), indicating that thrombolysis within 6 hours after ACI could suppress apoptotic molecule expression to alleviate cell apoptosis, which was consistent with the previous studies showing the beneficial role of rt‐PA in the control of neuronal death following stroke.^31^, ^32^

Acute cerebral infarction patients receiving thrombolytic treatment are at risk of some complications, including neural and vascular cell death, damage to the blood‐brain barrier, and intracerebral haemorrhage. Intracerebral haemorrhage is the most dangerous complication of thrombolysis, and its morbidity varies from 5% to 10%.^33^ In our study, the morbidity of intracerebral haemorrhage was 8.3% in the thrombolytic group, indicating that there was no significant difference when compared with previous studies.

There are several issues that need to be addressed. First, despite the present study and some clinical studies have suggested the efficacy and safety of thrombolysis beyond the therapeutic window of 4.5 hours,^34^ no change has been made in the officially approved therapeutic window. In any case, it is still believed that the earlier the treatment the larger the treatment benefits. Furthermore, some recent clinical trials have shown the efficacy and safety of endovascular treatment as a bridging therapy of thrombolysis in the treatment of acute stroke.^35^, ^36^ Therefore, future studies on clarifying the underlying mechanisms behind the effect of reperfusion/revascularization therapy on ACI should also take into account the thrombolysis treatment combined with endovascular therapy. Finally, there are many different experimental models of cerebral ischaemia.^37^ Because of the simplicity of the model, one model is insufficient to mimic all the phenotypes of clinical patients. Therefore, it would be interesting to perform the same analyses of this study using different experimental models of cerebral ischaemia.

In conclusion, thrombolytic therapy with rt‐PA within a broadened therapeutic window (6 hours) could significantly decrease the infarct volume after ACI, possibly by increasing the MVD, by decreasing the apoptotic molecule expression, and by decreasing free radical activities and enhancing SOD activities to alleviate the oxidative stress response in the ischaemic region. These findings provide a reliable theory basis for the clinical treatment of ACI with thrombolytic therapy within a broadened therapeutic window.

## ACKNOWLEDGEMENTS

This study was supported by grants from the Science and Technology Development Program of Shandong Province, China (no. 2011GGB14095), Medicine and Health Science Technology Development Program of Shandong Province, China (no. 2011HD009), Traditional Chinese Medicine Science and Technology Development Program of Shandong Province, China (2011‐194, 2017‐163), Natural Science Foundation of Shandong Province, China (no. Y2007C168, no. ZR2011HL020, no. ZR2012HL28, no. ZR2016HP23) and Key Science and Technology Program of Jinan City, China (201704113).

## CONFLICT OF INTEREST

The authors declare no competing or financial interests.

## References

[1] Bonita R , Mendis S , Truelsen T , Bogousslavsky J , Toole J , Yatsu F . The global stroke initiative. Lancet Neurol. 2004;3:391‐393. 15207791 10.1016/S1474-4422(04)00800-2

[2] Tissue plasminogen activator for acute ischemic stroke . The National Institute of Neurological Disorders and Stroke rt‐PA Stroke Study Group. N Engl J Med. 1995;333:1581‐1587. 7477192 10.1056/NEJM199512143332401

[3] Balami JS , Hadley G , Sutherland BA , Karbalai H , Buchan AM . The exact science of stroke thrombolysis and the quiet art of patient selection. Brain. 2013;136:3528‐3553. 24038074 10.1093/brain/awt201

[4] Robinson T , Zaheer Z , Mistri AK . Thrombolysis in acute ischaemic stroke: an update. Therap Adv Chronic Dis. 2011;2:119‐131. 23251746 10.1177/2040622310394032 PMC3513874

[5] Ogata T , Christensen S , Nagakane Y , et al. The effects of alteplase 3 to 6 hours after stroke in the EPITHET‐DEFUSE combined dataset: post hoc case‐control study. Stroke. 2013;44:87‐93. 23250996 10.1161/STROKEAHA.112.668301

[6] Hacke W , Furlan AJ , Al‐Rawi Y , et al. Intravenous desmoteplase in patients with acute ischaemic stroke selected by MRI perfusion‐diffusion weighted imaging or perfusion CT (DIAS‐2): a prospective, randomised, double‐blind, placebo‐controlled study. Lancet Neurol. 2009;8:141‐150. 19097942 10.1016/S1474-4422(08)70267-9 PMC2730486

[7] Ding YH , Li J , Zhou Y , Rafols JA , Clark JC , Ding Y . Cerebral angiogenesis and expression of angiogenic factors in aging rats after exercise. Curr Neurovasc Res. 2006;3:15‐23. 16472122 10.2174/156720206775541787

[8] Allen CL , Bayraktutan U . Oxidative stress and its role in the pathogenesis of ischaemic stroke. Int J Stroke. 2009;4:461‐470. 19930058 10.1111/j.1747-4949.2009.00387.x

[9] Chauhan A , Sharma U , Jagannathan NR , Reeta KH , Gupta YK . Rapamycin protects against middle cerebral artery occlusion induced focal cerebral ischemia in rats. Behav Brain Res. 2011;225:603‐609. 21903138 10.1016/j.bbr.2011.08.035

[10] Doyle KP , Simon RP , Stenzel‐Poore MP . Mechanisms of ischemic brain damage. Neuropharmacology. 2008;55:310‐318. 18308346 10.1016/j.neuropharm.2008.01.005 PMC2603601

[11] Choi‐Kwon S , Park KA , Lee HJ , et al. Temporal changes in cerebral antioxidant enzyme activities after ischemia and reperfusion in a rat focal brain ischemia model: effect of dietary fish oil. Brain Res Dev Brain Res. 2004;152:11‐18. 15283990 10.1016/j.devbrainres.2004.05.004

[12] Irmak MK , Fadillioglu E , Sogut S , et al. Effects of caffeic acid phenethyl ester and alpha‐tocopherol on reperfusion injury in rat brain. Cell Biochem Funct. 2003;21:283‐289. 12910483 10.1002/cbf.1024

[13] Heeba GH , El‐Hanafy AA . Nebivolol regulates eNOS and iNOS expressions and alleviates oxidative stress in cerebral ischemia/reperfusion injury in rats. Life Sci. 2012;90:388‐395. 22226906 10.1016/j.lfs.2011.12.001

[14] He S , Yang J , Wu B , et al. Neuroprotective effect of parthenocissin A, a natural antioxidant and free radical scavenger, in focal cerebral ischemia of rats. Phytother Res. 2010;24(Suppl 1):S63‐S70. 19565467 10.1002/ptr.2904

[15] Gaur V , Aggarwal A , Kumar A . Protective effect of naringin against ischemic reperfusion cerebral injury: possible neurobehavioral, biochemical and cellular alterations in rat brain. Eur J Pharmacol. 2009;616:147‐154. 19577560 10.1016/j.ejphar.2009.06.056

[16] Belniak‐Legiec E , Stelmasiak Z . Blood platelet activation markers in patients with acute cerebral infarction during the earliest stage of the disease–evaluation using flow cytometry methods. Neurol Neurochir Pol. 2000;34:853‐864. 11253475

[17] Kawiak W , Nowicka‐Tarach B , Gieracz A . [Levels of ammonia in patients with stroke]. Neurol Neurochir Pol. 1978;12:35‐38. 634430

[18] Pilarczyk M , Krasinska‐Czerlunczakiewicz H , Stelmasiak Z . Evaluation of lactic acid levels in blood of patients with ischemic stroke in the earliest stage of the disease. Neurol Neurochir Pol. 1999;32(Suppl 6):109‐111. 11107572

[19] Weglewski A , Ryglewicz D , Mular A , Jurynczyk J . [Changes of protein S100B serum concentration during ischemic and hemorrhagic stroke in relation to the volume of stroke lesion]. Neurol Neurochir Pol. 2005;39:310‐317. 16096936

[20] Overgaard K . The effects of citicoline on acute ischemic stroke: a review. J Stroke Cerebrovasc Dis. 2014;23:1764‐1769. 24739589 10.1016/j.jstrokecerebrovasdis.2014.01.020

[21] Longa EZ , Weinstein PR , Carlson S , Cummins R . Reversible middle cerebral artery occlusion without craniectomy in rats. Stroke. 1989;20:84‐91. 2643202 10.1161/01.str.20.1.84

[22] Thomalla G , Fiebach JB , Ostergaard L , et al. A multicenter, randomized, double‐blind, placebo‐controlled trial to test efficacy and safety of magnetic resonance imaging‐based thrombolysis in wake‐up stroke (WAKE‐UP). Int J Stroke. 2014;9:829‐836. 23490032 10.1111/ijs.12011

[23] Davis SM , Donnan GA , Parsons MW , et al. Effects of alteplase beyond 3 h after stroke in the Echoplanar Imaging Thrombolytic Evaluation Trial (EPITHET): a placebo‐controlled randomised trial. Lancet Neurol. 2008;7:299‐309. 18296121 10.1016/S1474-4422(08)70044-9

[24] Xie CY , Li CY , Yang J . Effect of mild hypothermia on cerebral infarct volume and angiogenesis in rats after cerebral ischemia. Med J Wuhan Univ. 2007;28:200‐202.

[25] Liu DH , Yuan FG , Hu SQ , et al. Endogenous nitric oxide induces activation of apoptosis signal‐regulating kinase 1 via S‐nitrosylation in rat hippocampus during cerebral ischemia‐reperfusion. Neuroscience. 2013;229:36‐48. 23137546 10.1016/j.neuroscience.2012.10.055

[26] Donnan GA , Davis SM . IST‐3: a major contribution to thrombolysis research. Int J Stroke. 2012;7:566‐567. 22989392 10.1111/j.1747-4949.2012.00938.x

[27] Donnan GA , Davis SM . Stroke: expanded indications for stroke thrombolysis–what next? Nat Rev Neurol. 2012;8:482‐483. 22847384 10.1038/nrneurol.2012.151

[28] Goto K , Ishige A , Sekiguchi K , et al. Effects of cycloheximide on delayed neuronal death in rat hippocampus. Brain Res. 1990;534:299‐302. 2073592 10.1016/0006-8993(90)90144-z

[29] Broughton BR , Reutens DC , Sobey CG . Apoptotic mechanisms after cerebral ischemia. Stroke. 2009;40:e331‐e339. 19182083 10.1161/STROKEAHA.108.531632

[30] Trapp T , Korhonen L , Besselmann M , Martinez R , Mercer EA , Lindholm D . Transgenic mice overexpressing XIAP in neurons show better outcome after transient cerebral ischemia. Mol Cell Neurosci. 2003;23:302‐313. 12812761 10.1016/s1044-7431(03)00013-7

[31] Benchenane K , Lopez‐Atalaya JP , Fernandez‐Monreal M , Touzani O , Vivien D . Equivocal roles of tissue‐type plasminogen activator in stroke‐induced injury. Trends Neurosci. 2004;27:155‐160. 15036881 10.1016/j.tins.2003.12.011

[32] Kim YH , Park JH , Hong SH , Koh JY . Nonproteolytic neuroprotection by human recombinant tissue plasminogen activator. Science (New York, NY). 1999;284:647‐650. 10.1126/science.284.5414.647 10213688

[33] Donnan GA , Davis SM , Parsons MW , Ma H , Dewey HM , Howells DW . How to make better use of thrombolytic therapy in acute ischemic stroke. Nat Rev Neurol. 2011;7:400‐409. 21670766 10.1038/nrneurol.2011.89

[34] Sandercock P , Wardlaw JM , Lindley RI , et al. The benefits and harms of intravenous thrombolysis with recombinant tissue plasminogen activator within 6 h of acute ischaemic stroke (the third international stroke trial [IST‐3]): a randomised controlled trial. Lancet (London, England). 2012;379:2352‐2363. 10.1016/S0140-6736(12)60768-5 PMC3386495 22632908

[35] Maier IL , Behme D , Schnieder M , et al. Bridging‐therapy with intravenous recombinant tissue plasminogen activator improves functional outcome in patients with endovascular treatment in acute stroke. J Neurol Sci. 2017;372:300‐304. 28017233 10.1016/j.jns.2016.12.001

[36] Kass‐Hout T , Kass‐Hout O , Mokin M , et al. Is bridging with intravenous thrombolysis of any benefit in endovascular therapy for acute ischemic stroke? World Neurosurg. 2014;82:e453‐e458. 23376392 10.1016/j.wneu.2013.01.097

[37] Prieto‐Arribas R , Moreno‐Gutierrez A , Simal‐Hernandez P , et al. [Experimental models of cerebral ischemia]. Revista de neurologia. 2008;47:414‐426. 18937203

